# Development of a novel conceptual framework for curriculum design in Canadian postgraduate trauma training

**DOI:** 10.36834/cmej.68621

**Published:** 2020-03-16

**Authors:** Brett Mador, Michael Kim, Jonathan White, Ilene Harris, Ara Tekian

**Affiliations:** 1 Department of Surgery, University of Alberta, Alberta, Canada; 2 Department of Medical Education, University of Illinois Chicago College of Medicine, Illinois, USA

## Abstract

**Background:**

Recent changes in practice patterns and training paradigms in trauma care have resulted in a critical review of postgraduate curricula. Specifically, a shift towards non-operative management of traumatic injuries, and reduced resident work-hours, has led to a significant decrease in trainees' surgical exposure to trauma. The purpose of our study is to perform an exploratory review and needs assessment of trauma curricula for general surgery residents in Canada.

**Methods:**

Our study design includes semi-structured interviews with trauma education experts across Canada and focus groups with various stakeholder groups. We performed qualitative analysis of comments, with two independent reviewers, using inductive thematic analysis to identify themes and sub-themes.

**Results:**

We interviewed four trauma education experts and conducted four focus groups. We formulated two main themes: institutional context and transferability of curricular components. We further broke down institutional context into sub-themes of culture, resources, trauma system, and trauma volume. We developed a new conceptual framework to guide ongoing curricular reform for trauma care within the context of general surgery training.

**Conclusions:**

The proposed framework, developed through qualitative analysis, can be utilized in a collaborative fashion in the curricular reform process of trauma care training in Canada.

## Abstract (fr)

**Contexte:**

Les changements récents liés aux modes de pratique et aux paradigmes de formation en matière de soins en traumatologie ont donné lieu à un examen critique des programmesde formation médicale postdoctorae. Précisément, la transition vers la gestion non chirurgicaledes blessurestraumatiques, et la réduction des heures de travail des résidents a diminué considérablement l’exposition chirurgicale des stagiaires aux situations de traumatologie. Le but de notre étude consiste à mener une revueexploratoire et une évaluation des besoins des programmes de traumatologie pour les résidentsen chirurgie générale au Canada.

**Méthodes:**

Notre devis comprend des entrevues semi-structurées auprès d’experts de formation en traumatologie de partout au Canada ainsi que des groupes de discussion avec différentes parties prenantes. Nous avons effectué une analyse qualitative des commentaires, avec deux examinateurs indépendants, au moyen d’une analyse thématique inductive pour définir des thèmes et sous-thèmes.

**Résultats:**

Nous avons interviewé quatre experts de formation en traumatologie et mené quatre groupes de discussion. Nous avons formulé deux thèmes principaux : le contexte institutionnel et la transférabilité des composantes du cursus. Nous avons également décomposé le contexte institutionnel en sous-thèmes de culture, de ressources, de systèmes de traumatologie, et de volume de traumatologie. Nous avons développéun nouveau cadre conceptuel pour guider les réformes en coursdes cursusde formation en traumatologie pour la formation en chirurgie générale.

**Conclusions:**

Le cadre proposé, et créé d’après une analyse qualitative, peut être utilisé de façon collaborative dans le processus de réforme du des cursus de formation en traumatologie au Canada.

## Introduction

Providing adequate education in major trauma care has become an increasingly challenging task for medical educators around the world. In developed countries we have seen a significant decrease in overall trauma volume, combined with a paradigm shift towards non-operative trauma management.^1^ Training standards have been further challenged by patient safety concerns and a focus on resident wellness, resulting in reduced trainee work hours and responsibilities.^2^^,^^3^ In parallel, the needs of the trauma trainee have expanded due to a steady increase in knowledge and technology related to trauma care.^4^

This widening gap between training needs and existing curricula, coupled with the unique contextual challenges of a widely distributed Canadian population,^5^ have led to calls for curricular reform.^6^ Kern describes a systems-based approach to curriculum design in health professions education, which begins with problem identification and a general and targeted needs assessment.^7^ The purpose of this study is to perform an exploratory study and initial needs assessment, in order to create a framework for ongoing curricular reform of trauma care training in Canada.

## Methods

Our study was approved by the institutional review boards at both the University of Alberta and the University of Illinois, Chicago, and we obtained informed consent from all participants. We prepared an initial needs assessment semi-structured interview guide, using conceptual frameworks described by Kern^7^ and Lee et al.^8^ We performed a comprehensive review of existing trauma curricula across Canada, including reviews of published literature, available curricular documents, and perspectives of interview participants.

We piloted our initial interview guide twice on general surgeons, with iterative changes, and revised it based on feedback. We then used purposive sampling to identify trauma experts (general surgeons with specific training in trauma care) from tertiary trauma centres across Canada, selecting lead trauma educators and avoiding duplicate participants from the same city or program. We used snowball sampling, using known contacts, to form a list of potential participants whom we recruited by e-mail, continuing until we had identified at least one potential participant from each English-speaking trauma centre. This sampling strategy focused on specific, high yield participants from diverse environments. We conducted 30 to 60 minute semi-structured interviews using open-ended questions. We acknowledged reflexivity, based on the interviewer's trauma experience and biases.

We took a constructivist approach to the data analysis,^9^ utilizing inductive thematic analysis by two independent reviewers. Both reviewers are trauma surgeons with training in qualitative analysis. We conducted thematic analysis, following well-described stages, including immersion in the data, formulation of themes and sub-themes, and revision of themes based on discussion until reaching consensus.^10^ We achieved theoretical saturation by increasing the number of interviews until no further major themes were formulated with subsequent data. Finally, we achieved consensus between reviewers, resulting in selected themes and sub-themes which we used to develop a focus group guide.

We recruited focus group participants by e-mail from a single general surgery residency program. We split planned focus groups of two to six participants into junior trainees (first and second year), senior trainees, and trauma surgeons. We added a fourth group based on analyzed interview data, consisting of general surgeons working at non-trauma designated community training sites. We accomplished triangulation by including multiple data collection methods and using multiple participant groups. We performed focus group data collection and analysis similarly to the interview data. We used NVivo 11 (QSR International, Doncaster, Australia) for the organization of the qualitative data. We organized the consensus-based final thematic map into a framework for the purpose of guiding future curriculum development.

## Results

**Figure 1:**
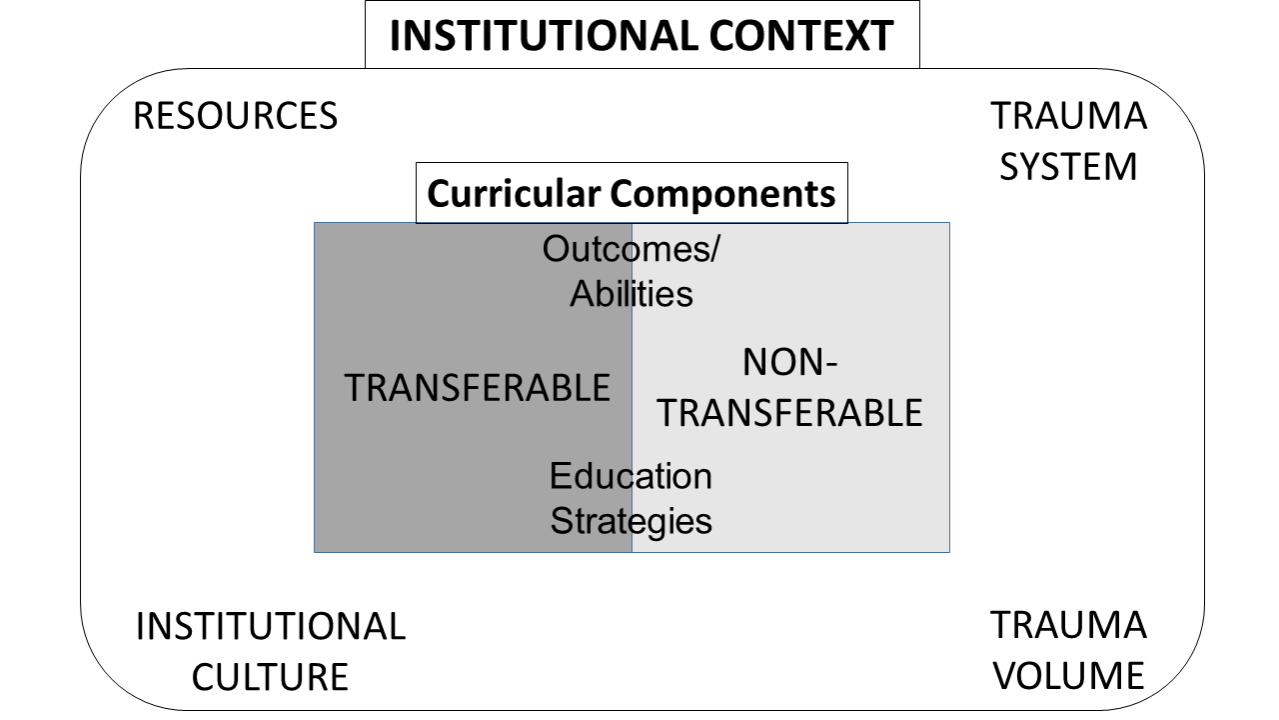
Conceptual framework for curriculum development in trauma care

We conducted four semi-structured interviews with trauma experts, followed by four focus groups, with six participants in the junior resident group, five in the senior resident group, and two each in the trauma educator and community surgeon groups. Final consensus resulted in two major themes: institutional context and transferability of curricular components. We then split transferability into two sub-theme categories: transferable and non-transferable, each of which contain multiple curricular components, including trainee outcomes and education strategies. We split the theme of institutional context into four specific, but interconnected, sub-themes: institutional culture, resources, trauma systems and trauma volume. We created a conceptual framework (Figure 1) to display the relationships among the various themes and sub-themes. Illustrative quotations from the text, supporting each theme, are displayed in Table 1.

**Table 1:** Themes and sub-themes with illustrative quotations from the data

Theme	Illustrative Quotations
**Institutional context**	
Institutional culture	*“I think if you build a really robust program that engages the learners at a junior level then they are going to, [at] senior levels, by default they are going to come in for these trauma [cases]. The problem is I feel with the current climate the residents have never really had strong trauma education and so I get comments like can your [nurse practitioner] come down for the resuscitation or I can't come in for trauma team activations because of sleep hygiene issues.” -Educator* *“The thought process that this [view of trauma care] might be shifting a little bit but let’s just say five years ago, everyone will say I am a general surgeon, trauma is nothing special, we can do trauma. Now there is a shift…but we really need champions to continue to exert steady pressure in multiple ways to push us along, because the culture has never been trauma focused.” -Educator*
Resources	*“Trauma surgery training can also be very resource heavy which is potentially why maybe it gets put on the back burner sometimes. If you want to have a dedicated trauma service and trauma surgeons to do the education you [have] got to hire them which is at the cost of potentially other people. If you want to run [simulations] on pigs or mannequins you have to buy those at the cost of other people. So, I think sometimes that becomes a barrier because potentially people may not see it as a priority.”* *-Trainee* *“The availability and infrastructure plays an important role.” -Educator*
Trauma system	*“I think just having dedicated trauma makes it even better, like having dedicated trauma surgeons who are up-to-date and know everything about it…and having [a] dedicated trauma service and uniform care for trauma patients” -Trainee* *“Coming from a different training program we lack that, the ability or the opportunities for regular involvement, leading the team or making some decisions.”-Educator*
Trauma volume	*“You can't schedule your trauma.” -Trainee* *“Canada wide volume could be an issue because it's inconsistent in different programs for sure.” -Trainee* *“So, they understood the concepts and how they should approach the injuries from a book perspective but they don't have any of the real world exposure in terms of [resuscitating] the patient at [the] trauma bay as well as taking care of the kind of non-classical general surgery aspects of trauma care postoperatively.” -Educator* *“I think it is really crazy how rare operative trauma is becoming…It almost seems like a commercial on TV for this species [that] is going extinct, donate now, save trauma surgeons, [they] won't have a population.”* *-Educator*
**Curricular components**	
Transferability	*“I still haven't done that much operative trauma. However, now that I've finished my chief year and I've done enough ORs I do feel comfortable with a lot of the operative maneuvers. I just haven't done them in the trauma setting.” -Trainee* *“You just keep talking about the operative management and what you would do if you were in that situation, [it] becomes much more valuable because then once you know those skills as a chief and you are like, I have those – I know those maneuvers we can then apply them appropriately.”* *-Trainee* *“I think you can get a lot of the skills, the basic skills from non-trauma stuff but need to be able to translate them into high energy situation[s].”* *-Educator*
Trainee outcomes	*“I think it's tough [in] trauma how much you should know and what your skill set should be; there is airway, there is access, there is surgery, thoracotomy, there is chest tubes and then being the [trauma team leader] or [doing] the primary or secondary [survey] and it's tough to say that should you be competent in all of those, [and] how competent should you be?” -Trainee* *“I think things that get neglected might be…non-abdominal operations. I worry that they would be completely lost in the neck, in the extremities and certainly vascular exposures.” -Educator* *“The resuscitation part of it and the decision making, that’s the hard part.” -Educator*
Education strategies	*“Right now, I am not sure that we have a good understanding of what the curriculum for trauma actually looks like.” -Trainee*
Teaching	*“I would also say I think they have to be good teachers, and by that I mean they have to engage the group.”-Trainee* *“Doing things with supervision and getting feedback, that's really what it's all about, whether that’s a real trauma scenario or simulation.” -Educator*
Assessment	*“We are all under the microscope all the time.” -Educator* *“The easiest things to assess is in the [operating room], how they take out the spleen, you can give them move by move feedback…There is not going to be one standardized assessment way, you can do [it] as a variety; there is feedback, there is coaching, there is [in-training evaluation reports] and I am sure the [competency-based] thing will come along.” -Educator* *“I think the most reliable metric of a clinician’s competence is the judgment of experienced educators. You know when your junior resident is flustered and you know when your senor resident gets it, you know it in your guts, like you just know.” -Educator*
Planned education activities	*“I like having mandatory standardized courses like ATLS (Advanced Trauma Life Support) and ATOM (Advanced Trauma Operative Management) involved in residents’ curriculum because I feel like it sets a certain bar with regards to the requirements that you need to achieve, with regards to resuscitation, patient care and operative damage control interventions.” -Trainee* *“I think there is a growing body of research and evidence to support simulation and that's evolving with time, and I think if you look at the direction in which medicine is going, simulation is going to play an important role. So, I think it's absolutely key that we incorporate it into our training.” -Educator*

## Institutional context

All participants cited the institutional context, in some way, as having a substantial impact on trauma curricula. Educators discussed the specific trauma culture of the institution, the need for standardization across training programs, and the overall organization of the trauma system. Learners, on the other hand, are more focused on their individual learning environments, such as which type of physicians are leading the trauma resuscitations, how residents are integrated into the trauma system, and the volume of cases to which learners are exposed and have experience.

### Institutional culture

The culture related to trauma is viewed by participants as impacting curricular content, instruction, and learner achievement. Specifically, a strong trauma culture can create an engagement in trauma care that filters from staff surgeons through to the medical student level. Trauma champions, such as trauma-trained surgeons, are perceived to bolster positive culture change through their engagement and commitment to holistic trauma care.

### Resources

When discussing challenges in trauma curricula, available resources, including their utilization and funding, are frequently cited as major issues. From a broad perspective, resources to build and maintain the trauma system itself, such as hiring additional trauma-specific personnel or capacity building, indirectly affects the training environment. Resources are also required for ancillary training opportunities such as simulation and trauma courses.

### Trauma system

The loco-regional trauma system provides the background in which clinical learning takes place. In most programs it is mandated that trainees spend time at an academic trauma center, but even within this context, trainee experiences can vary considerably. For example, smaller centres tend to have less surgical trauma team leaders for resuscitation-based training, a lack of inpatient trauma services, and inconsistent surgical trainee involvement in major trauma care.

### Trauma volume

Adequate case volume is cited by both learners and educators as a critical aspect of trauma training, in addition to the use of complementary education approaches such as simulation. The variability in these experiences at different programs across Canada, and even within programs, is considered to be a major threat to consistently training trauma-competent surgeons.

## Curricular components

A large proportion of the discourse was devoted to the defined, planned portions of the delivered curricula, specifically trainee outcomes (i.e., achievement of learning goals and objectives) and education strategies (i.e., teaching, learning, and assessment).

### Transferability

The concept of transferability was identified frequently during discussions about curricular components, i.e., what skills do I need specifically from the trauma curricula and what skills can I learn electively and apply when needed. However, transferability needs to be viewed within the overarching theme of context. For example, performing a laparotomy for trauma as compared to oncology, while utilizing multiple transferable components (i.e., anatomy knowledge, surgical skills), is still not fully transferable due to inherent non-transferable aspects, such as operating in the presence of major bleeding and the need for enhanced situational awareness.

### Trainee outcomes

Outcomes of interest from our data are non-technical skills, including leadership, teamwork and decision-making. These skills, specifically in the context of trauma resuscitation, are viewed by both trainees and educators to be crucial, yet underemphasized, in the curriculum. Operative competence is also considered important for trainee learning and outcomes, but with the exception of certain rare operations and non-abdominal surgery, the majority of operative skills are considered transferable from elective training.

### Education strategies

Education strategies are used here to describe all aspects of planned teaching, learning and assessment. The study participants identified a need to determine what exactly is included in the curriculum, and supported the use of national standardization. Based on participant data, education strategies are further classified in three sub-themes: teaching, assessment, and planned education activities. The need for dedicated trauma-focused surgeon teachers is emphasized by trainees and educators alike as being foundational to optimal trauma care training. In terms of assessment, participants stressed the importance of frequent observations with feedback, often citing the benefits of a competency-based education framework. Finally, different types of instructional methods are discussed in detail, with strong support for the necessity of specific trauma courses and simulation.

## Discussion

Consistent with existing literature, institutional context is perceived by our study participants to play a major role in trauma curricula.^11^^-^^13^ In terms of curricular design, however, it is most helpful to focus on the readily modifiable aspects. For example, resources can be re-allocated, specifically to support trauma-specific courses that are supported both by our data and by existing education research.^14^^-^^18^ Further, trauma system improvement is also viewed as bolstering trauma education, in addition to having proven patient-level benefits in terms of trauma-centre accreditation,^19^ and dedicated inpatient trauma services.^20^

Trauma volume, however, is the most difficult barrier to overcome. While from a societal perspective this decline in volume is very positive, it has produced unique training challenges, as the training standards have not decreased. Further, even when trauma does present it often does not require surgical management and may not even have involvement of the surgical trainee. The option of sending all trainees for elective rotations in high trauma areas is not feasible. However, alternative solutions exist. Trauma volume per trainee can be optimized via deliberate trainee scheduling,^21^^,^^22^ focusing on the busier times of the day or the year, and use of a competency-based curricular model to minimize variability. These modifications can be further supplemented by increased use of simulation, both resuscitative and operative, and curricular innovation.

The other major theme that relates to curricular design is the concept of transferability, which has been defined as, “the extent to which the measured effectiveness of an applicable intervention could be achieved in another setting.”^23^ Our results indicate that operative skills are perceived to be particularly transferable from the elective setting to trauma care. Given current challenges in achieving trainee operative trauma volumes, this provides a key tool for curricular planners to fill needed gaps in methods of achieving trainee competence.^1^^,^^4^ This includes both training in supplementary areas (i.e. thoracic surgery, elective neck surgery) and also simulation.^24^ In addition to technical skills, transferability also applies to important curricular elements, such as teaching strategies and broad frameworks such as competency-based education.

This study has multiple limitations. The participation rate for the surgeon focus groups is poor, despite providing flexibility for participants in timing and location. Our study is also limited by the use of just one training program for participant selection for focus groups. Given the focus of the study on the Canadian context, its generalizability to other countries is uncertain.

### Conclusion

Given a background of previously described deficiencies in general surgery trauma training, our study presents a framework for the curricular design and reform process of trauma care training. Building on previous work, we feel that our new framework provides a foundation for ongoing curricular review, including a more robust needs assessment utilizing mixed methods, and eventually consensus-based decision-making methods to formulate new training guidelines. Collaboration is required between the individual programs across Canada, and the accrediting bodies, to create a standardized, national curriculum to adequately supplement existing competency-based training and provide needed trauma expertise to satisfy societal needs.
